# A rare case of recurrent primary anorectal melanoma emphasizing the importance of postoperative follow-ups

**DOI:** 10.1186/s12893-020-00727-6

**Published:** 2020-04-07

**Authors:** Zhihao Li, Peter Šandera, Marc Beer, Markus Weber

**Affiliations:** 1 Department of Visceral, Thoracic, Vascular Surgery, Municipal Hospital Triemli, Birmensdorferstrasse 497, 8063 Zürich, Switzerland; 2 Department of Pathology, Municipal Hospital Triemli, Birmensdorferstrasse 497, 8063 Zürich, Switzerland

**Keywords:** Abdominoperineal resection, Anorectal mass, Postoperative follow-ups, Primary anorectal melanoma, Wide local excision

## Abstract

**Background:**

Primary anorectal melanoma can be a rare differential diagnosis of anorectal mass. Due to the low case number reported in the literature, physicians are not aware of this aggressive disease. Although no consensus exists, wide local excision and abdominoperineal resection are considered the mainstay therapy.

**Case presentation:**

An 85-year-old female patient presented with fecal incontinence 5 years after local resection of a primary anorectal melanoma. In the rectoscopy, a tumor proximal to the dentate line was identified and later confirmed as a recurrent primary anorectal melanoma. There were no signs of locoregional or distant metastasis on the MRI and PET/CT. She underwent another wide local excision and regained fecal continence postoperatively.

**Conclusions:**

Primary anorectal melanoma should belong to the differential diagnosis of anorectal mass. If technically feasible, wide local excision represents a less invasive treatment than abdominoperineal resection, retaining the anal sphincter and patient’s quality of life. Even though wide local excision has a higher recurrence rate than abdominoperineal resection, there is no difference in survival between the two procedures. This is only under the premise that patients are followed-up regularly after wide local excision so that recurrence can be spotted early on and locally excised.

## Background

Primary anorectal melanoma (PARM) is an extremely rare but highly aggressive disease. It accounts for 0.5 to 2% of all anorectal malignancies and less than 2% of all melanomas [1]. PARM presents in the fifth or sixth decade of life, predominantly in women. The most common presenting complaints of patients are bleeding, anal pain, pruritus, tenesmus, and change in bowel habits. The lesions are usually polypoid, lack pigmentation in up to 80% of the cases and are often mistaken for hemorrhoids or polyps leading to delayed diagnosis. PARM must be differentiated from metastasis of a primary cutaneous melanoma to the anorectum, which constitutes 0.04% of all cutaneous melanoma metastases [1]. This distinction can be difficult, since 26–38% of patients with PARM already have metastatic diseases at the time of diagnosis [2, 3]. Even though there is no consensus due to the rarity of this entity, surgical removal (wide local excision or abdominoperineal resection) is considered the mainstay of treatment.

## Case presentation

We report a local recurrence of PARM in an 85-year-old female patient 5 years after the resection of the primary tumor.

Five years ago, the patient presented with fecal incontinence, anal pruritus and bleeding. A polypoid structure above the dentate line at 4 o’clock position was identified in the rectoscopy. In the endosonography, the lesion appeared as an ulcer of 1 cm diameter infiltrating the rectum wall. The biopsy result surprisingly confirmed a rare anorectal melanoma. No metastases were identified on the PET/CT and a dermatological examination did not find any other primary cutaneous tumor sources. The tumor was locally excised with a R0-resection. It had a thickness of 0.22 cm. The patient was followed-up 6 months postoperatively by her general practitioner and a rectoscopy 3 years postoperatively was uneventful. Unfortunately, the follow-up was discontinued thereafter. She was referred to us again by her gynecologist who was treating her for persistent dermatitis of the vulva due to fecal incontinence with suspicion for local tumor recurrence.

**Fig. 1:**
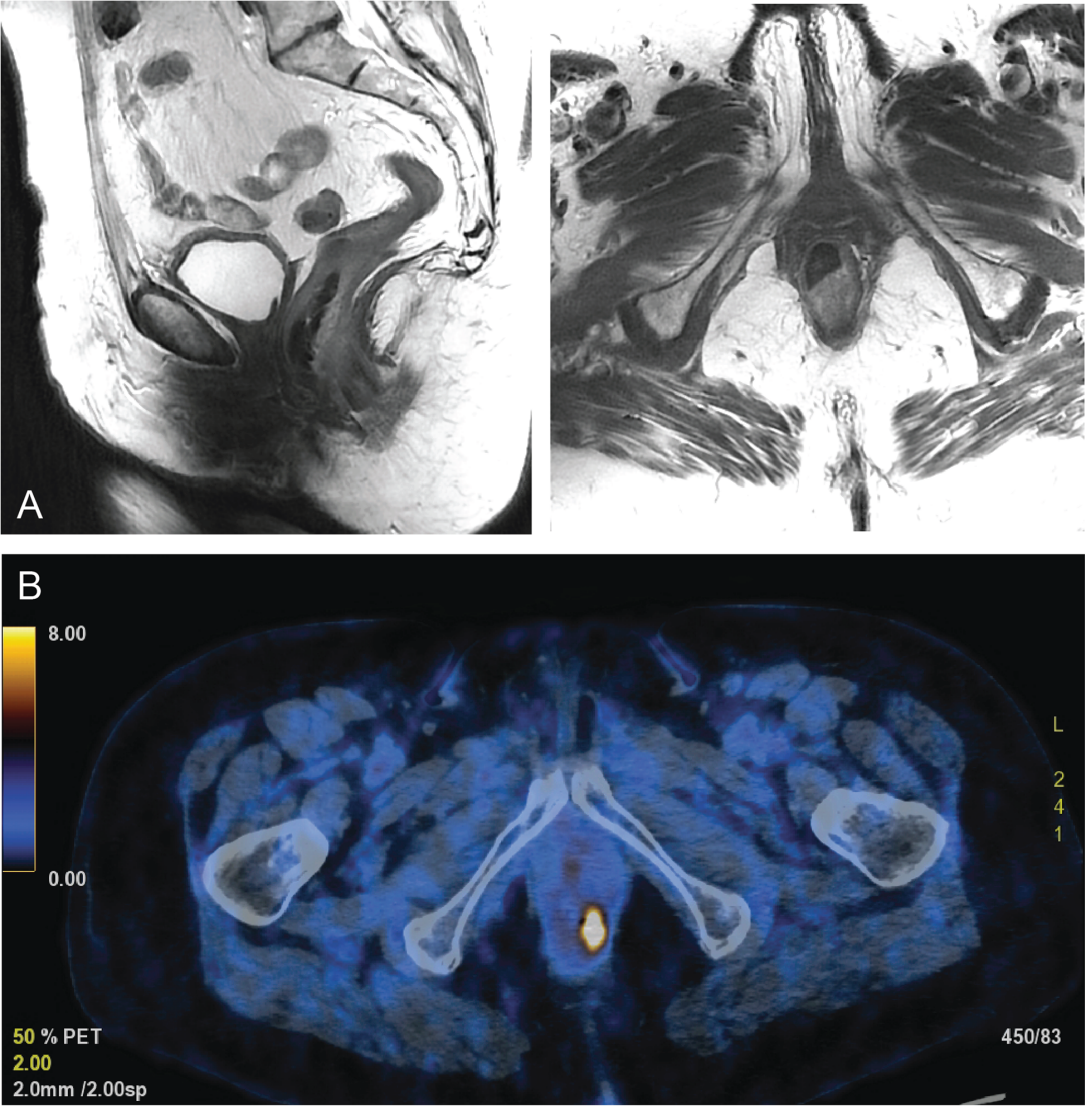
**a** MRI pelvis: 4 cm long thickening of the dorsal distal rectum wall without signs of sphincter infiltration; **b** 18F-FDG PET-CT: Metabolic active lesion at the anorectal transition without signs of lymphatic or distant metastasis

**Fig. 2:**
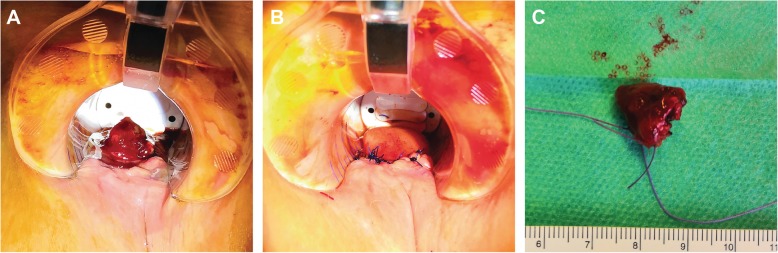
**a** Preoperative finding: the tumor presents as a polyp at 6 o’clock protruding into the anal canal; **b** Postoperative finding: the tumor is locally resected, and the mucosa defect closed; **c**) The 1 cm large resected tumor

**Fig. 3:**
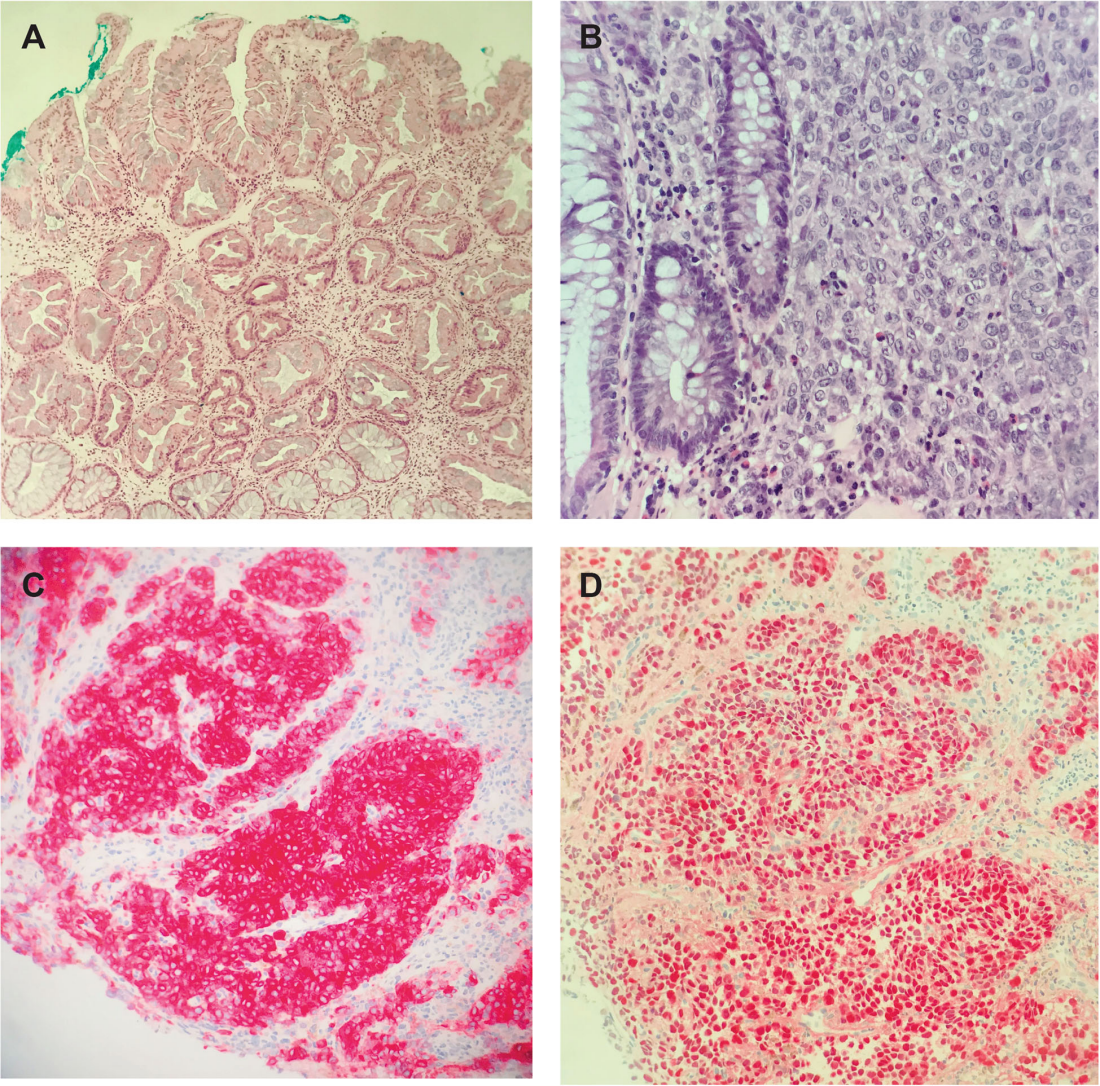
**a** HE stain, 100x. Colonic polyp with serrated configuration and basal crypt dilation, consistent with a sessile serrated lesion (formerly sessile serrated adenoma/polyp). No collision between the melanoma and the polyp was visible on any histologic slide. **b** HE stain, 400x. Diffuse infiltrates of melanoma cells with characteristic prominent nucleoli encroaching on benign colonic glands. **c** Positive immunostaining for Melan A and D) S100 confirms the melanocytic differentiation of the tumor

A new 1 cm large pigmented polyp at 6 o’clock, proximal to the dentate line, protruding into the anal canal was identified (see Fig. 2a). A punch biopsy of this lesion demonstrated malignant cells positive for tumor marker Melan A and S100 confirming the diagnosis of a recurrent malignant melanoma. The complete closure of the sphincter was obstructed due to the protrusion of the tumor into the sphincter apparatus explaining the fecal incontinence. A colonoscopy could not find any other pathologies and an MRI did not show any infiltration into the internal sphincter (see Fig. 1a). In the repeated PET/CT, an intensely hypermetabolic lesion centered on the lower rectum (standardized uptake value (SUV) = 16.2) was found without any evidence of regional lymph node or distant metastasis confirming a local recurrent Stage I tumor disease (see Fig. 1b). The multidisciplinary oncologic board decided to perform a WLE without adjuvant therapy. The operation was successful with an uneventful postoperative period (see Fig. 2). The histology examination confirmed a R0-resection and the rare concurrent finding of a sessile serrated adenoma (see Fig. 3). The tumor had a depth of 0.8 cm. The patient was asymptomatic after the surgery and was no more fecal incontinent. The preoperative Wexner score of 8 has been reduced to 2 postoperatively. According to the decision of the following oncologic board, the patient will be followed-up with physical examination and rectoscopy every 6 month in our pelvic floor unit.

## Discussion and conclusions

PARM is the second most common subtype of mucosal melanoma after ocular melanoma. It usually originates from the dentate line where melanocytes reside and can spread along the submucosal plane and drain to the inguinal and inferior mesenteric lymph nodes. At the time of diagnosis, one third of the patients have metastatic diseases beyond surgical cure and their five-year survival is only 3 to 22% [1]. This report describes a recurrence of anorectal melanoma without distant metastases 5 years after the initial WLE, which offers a beneficial prognosis.

While the staging of cutaneous melanoma is mainly based on the Breslow classification, PARM is only classified on disease spread. Local disease is described as stage I, regional lymph node disease as stage II, and metastatic disease as stage III. Since mucosal melanoma typically presents a nodular growth pattern, the Breslow depth for cutaneous melanoma is not always applicable to mucosal melanomas. It is controversial if the tumor size and thickness are prognostic factors. Thibault et al. reported that patients with a tumor thickness ≤ 2 mm have better survival than patients with lesions > 2 mm [2]. Goldman et al. [4] found a correlation between overall survival and tumor size, showing greater overall survival for patients with tumors ≤2 cm. In more recent studies however, tumor size and thickness were not associated with disease-specific survival [5, 6]. The thickness of the primary tumor of this case was 0.22 cm, while the recurrent tumor was 0.8 cm, far above the 2 mm threshold. Nevertheless, the patient enjoyed a metastasis-free survival of 5 years so far.

Surgery is the mainstay treatment for PARM. The procedures include radical abdominoperineal resection (APR) and limited wide local excision (WLE). Due to the rarity of this disease, there is a lack of prospective or randomized data to provide evidence-based treatment recommendations. APR is associated with a wider resection including mesenteric lymphadenectomy and hence a better loco-regional tumor control and lower recurrence rate compared to WLE. However, there is no difference in survival between the two procedures meaning that the extent of surgical excision does not correlate with survival [5–7]. In addition, the more invasive APR is associated with high morbidity and permanent colostomy compared to WLE [1]. The inconvenience of having a colostomy can signify a loss in life quality for the patient. WLE has minimal morbidity and little compromise in the local function preserving the anal sphincter. Even though the local recurrence with WLE might be higher than APR (47% vs. 23% [8]), close surveillance and aggressive re-excision can control the tumor locally. Hence, WLE is recommended whenever negative margins can be achieved to maintain life quality of the patient. APR should be reserved for selected patients in whom WLE is not technically possible, i.e. due to tumor infiltration of the anal sphincter. Despite therapy, patients with only local disease survive 34 months and 13 months with regional spread [9].

In this case report, the 85-year-old patient was still in relatively good health and maintained an active lifestyle. She valued a high quality of life over a long disease-free life expectancy. Her wish is to become fecal continent again regaining her life quality with the least invasive surgery. Here, the MRI was a useful diagnostic tool to verify the involvement of the sphincter which had a relevant impact on the extend of surgery. The tumor in our case did not infiltrate the sphincter, which was the reason we agreed with the patient on performing another WLE instead of APR resection, maximizing her quality of life as well as providing the best chance of cure.

While sentinel lymph node biopsy and lymphadenectomy are essential in the treatment of cutaneous melanoma, it is not well established for PARM. Sentinel node biopsy and subsequent inguinal dissection is technically feasible detecting clinically unapparent groin adenopathy and providing a curative lymph node resection. However, the numbers are too small to support this treatment modality. The prognosis with and without complete lymph node dissection is not significantly better [5, 6]. In the absence of clinical signs of regional lymph node metastasis and patient’s preference for the least invasive procedure, we did not perform additional lymph node dissection.

This case illustrates the importance of close follow-ups after a WLE. Our patient was not routinely followed up after her first WLE hence the recurrent tumor could grow to a significant large size increasing the risk of metastasis. For cutaneous melanoma, guidelines for follow-ups exist. They are stage-specific and recommend history and physical examination at least annually [10]. Self-examination and patient education are important cornerstones in the follow-up surveillance. In comparison, no guidelines exist for anorectal melanoma due to the low case number. Self-examination is obviously not feasible since the lesions are not visible to the patient. Especially in the cases following WLE where the locoregional recurrence is higher than APR, patients should be routinely followed-up. We recommend physical examination with rectoscopy every 6 months in the first 2 years and then annually for life.

In summary, anorectal melanoma is a rare but aggressive disease with dismal prognosis. Compared to cutaneous melanoma, there is a lack of evidence-based guidelines regarding the treatment and follow-up for anorectal melanoma. In the process of defining the treatment strategy, the attending physician should not only aim for tumor clearance but also consider the patients’ preference which could be the improvement of their life quality. WLE can be seen as a less invasive treatment option with equal effectiveness compared to APR. However, due to high locoregional recurrence rate, close follow-ups are crucial for early detection and re-resection of the recurrent tumor to guarantee a metastasis-free survival of the patient.

## Data Availability

All data is contained within the manuscript file.

## Abbreviations

PARM: Primary anorectal melanoma
APR: Abdominoperineal resection
SUV: Standardized uptake value
WLE: Wide local excision
